# *Aspergillus fumigatus* Photobiology Illuminates the Marked Heterogeneity between Isolates

**DOI:** 10.1128/mBio.01517-16

**Published:** 2016-09-20

**Authors:** Kevin K. Fuller, Robert A. Cramer, Michael E. Zegans, Jay C. Dunlap, Jennifer J. Loros

**Affiliations:** a Department of Molecular and Systems Biology, Geisel School of Medicine at Dartmouth, Hanover, New Hampshire, USA; b Department of Microbiology and Immunology, Geisel School of Medicine at Dartmouth, Hanover, New Hampshire, USA; c Department of Surgery (Ophthalmology), Geisel School of Medicine at Dartmouth, Hanover, New Hampshire, USA; d Department of Biochemistry and Cell Biology, Geisel School of Medicine at Dartmouth, Hanover, New Hampshire, USA

## Abstract

The given strain of *Aspergillus fumigatus* under study varies across laboratories, ranging from a few widely used “standards,” e.g., Af293 or CEA10, to locally acquired isolates that may be unique to one investigator. Since experiments concerning physiology or gene function are seldom replicated by others, i.e., in a different *A. fumigatus* background, the extent to which behavioral heterogeneity exists within the species is poorly understood. As a proxy for assessing such intraspecies variability, we analyzed the light response of 15 *A. fumigatus* isolates and observed striking quantitative and qualitative heterogeneity among them. The majority of the isolates fell into one of two seemingly mutually exclusive groups: (i) “photopigmenters” that robustly accumulate hyphal melanin in the light and (ii) “photoconidiators” that induce sporulation in the light. These two distinct responses were both governed by the same upstream blue light receptor, LreA, indicating that a specific protein’s contribution can vary in a strain-dependent manner. Indeed, while LreA played no apparent role in regulating cell wall homeostasis in strain Af293, it was essential in that regard in strain CEA10. The manifest heterogeneity in the photoresponses led us to compare the virulence levels of selected isolates in a murine model; remarkably, the virulence did vary greatly, although not in a manner that correlated with their overt light response. Taken together, these data highlight the extent to which isolates of *A. fumigatus* can vary, with respect to both broad physiological characteristics (e.g., virulence and photoresponse) and specific protein functionality (e.g., LreA-dependent phenotypes).

## IMPORTANCE

The current picture of *Aspergillus fumigatus* biology is akin to a collage, patched together from data obtained from disparate “wild-type” strains. In a systematic assessment of 15 *A. fumigatus* isolates, we show that the species is highly heterogeneous with respect to its light response and virulence. Whereas some isolates accumulate pigments in light as previously reported with strain Af293, most induce sporulation which had not been previously observed. Other photoresponsive behaviors are also nonuniform, and phenotypes of identical gene deletants vary in a background-dependent manner. Moreover, the virulence of several selected isolates is highly variable in a mouse model and apparently does not track with any observed light response. Cumulatively, this work illuminates the fact that data obtained with a single *A. fumigatus* isolate are not necessarily predictive of the species as whole. Accordingly, researchers should be vigilant when making conclusions about their own work or when interpreting data from the literature.

## INTRODUCTION

Though fungi cannot harness light as a direct source of energy, many nevertheless utilize it as a source of information. For example, light may inform the fungus of its position in the environment such that spore release is optimally timed and directed toward the open air. Visible light can also serve as an indicator of cooccurring environmental stresses (e.g., UV radiation, elevated temperature, and desiccation), thereby promoting stress resistance and, consequently, survival (1–3). Indeed, photosensory pathways can promote fungal fitness in complex and poorly understood ways, as illustrated by the fact that loss of a conserved blue light receptor (the *wc-1* ortholog in *Neurospora crassa*) (4, 5) leads to attenuated virulence in the distantly related human pathogens *Cryptococcus neoformans* and *Fusarium oxysporum* (6, 7). Thus, a better understanding of fungal photobiology can have practical implications in all fields affected by these organisms, including agriculture, industry, and medicine.

The ascomycete mold *Aspergillus nidulans* has served as a premier model for understanding how fungi integrate multiple photosensory inputs. In this organism, for example, asexual development (conidiation) is additively induced by blue and red light through the action of discrete photoreceptor proteins LreA and FphA, respectively (8, 9). Recent insight into how these two proteins regulate the light response at the molecular level will be briefly discussed here.

LreA is a blue-light-sensing GATA-type transcription factor and ortholog of *Neurospora* White Collar-1 (WC-1) (4, 5). In *Neurospora*, WC-1 directly drives transcription of target genes following photoactivation, and consequently, *Δwc-1* mutants fail to accumulate conidia or carotenoids in the light (4, 5, 10). In contrast, the *ΔlreA* mutant of *A. nidulans* actually displays a slight increase in conidiation regardless of the light environment, suggesting that LreA serves as a general repressor of gene expression (8). Biochemical data support the genetic inference, as LreA directly interacts with the promoter of at least one light-induced gene (*ccgA*) in the dark and is subsequently released from the DNA in light (11). In addition to DNA, LreA directly interacts with the chromatin-modifying enzymes HdaA (histone deacetylase) and GcnE (histone acetylase); therefore, LreA-mediated repression is believed to, in part, involve chromatin modification at the promoter of target genes (11).

FphA is a red-light-sensing phytochrome that utilizes a conserved histidine kinase domain to regulate downstream components (12, 13). Deletion of *fphA* leads to an attenuated photoinduction of conidiation, indicating that FphA promotes the expression of asexual genes in light (8). This induction is currently modeled to occur in two ways: in the nucleus, light-activated FphA interacts with and promotes the activity of GcnE to promote histone acetylation and induction of target loci (11); in the cytoplasm, dark-state FphA represses the conidiation-inducing activity of the mitogen-activated protein (MAP) kinase SakA (HogA) (14).

In summary, light directly promotes asexual development in *A. nidulans* by simultaneously inhibiting the activity of the blue light receptor LreA (a repressor) and promoting the activity of the red light sensor FphA (an inducer). Interestingly, LreA and FphA interact physically within a nuclear complex that contains both LreB (the ortholog of WC-2 of *Neurospora*) and the developmental regulator VeA (Velvet) (8, 15). Although it is not a photoreceptor, an important role for VeA in regulating the light dependency of *A. nidulans* development has long been known; specifically, the *veA* mutants do not require light stimulus to undergo asexual differentiation and instead produce conidia robustly even in the dark (16–19). Moreover, the *ΔveA* mutants display partial derepression of *ccgA* expression and VeA interacts with the *ccgA* promoter in an FphA-dependent manner (11). Similarly to LreA and FphA, VeA also interacts with HdaA and GcnE (11). Taken together, VeA is currently modeled to serve as a transcriptional repressor of asexual development whose activity is negatively controlled by FphA.

Unlike the above story of *A. nidulans*, we recently reported that light does not have a significant influence over conidiation in the opportunistic pathogen *Aspergillus fumigatus* (20). The fungus does, however, contain orthologs to both LreA and FphA and is overtly responsive to light in other ways. First, *A. fumigatus* (strain Af293) intensely accumulates protective mycelial pigments (melanin) under white or blue light illumination, a response that is abolished in a *ΔlreA* mutant. Second, light negatively impacts both conidial germination kinetics and cell wall homeostasis, the latter determined by sensitivity to Congo red. Curiously, both the germination and cell wall phenotypes are under the control of the FphA ortholog, despite blue light playing a central role in mediating the effects. Taking the pigmentation and growth responses together, we concluded that light serves primarily as a stress signal for *A. fumigatus*, rather than a developmental cue. Indeed, a brief exposure of *A. fumigatus* to light enhanced resistance to subsequent exposure to either UV-B or hydrogen peroxide (20). A divergent response to light between *A. fumigatus* and *A. nidulans* is perhaps not surprising considering that the evolutionary divergence between them (based on average protein identity) is comparable to that of humans and fish (21). The disparity between the pathogenic potentials of the two aspergilli further illustrates this physiological divergence.

*A. fumigatus* also contains an ortholog of *veA*, and as in *A. nidulans*, it too plays a role in asexual development. Remarkably, however, the exact influence of VeA has been found to differ depending on the background strain being investigated. In strain Af293 (the strain used in our studies described above), a *ΔveA* mutant displays increased conidiation levels, similarly to the reports in *A. nidulans* (22); in contrast, conidiation is reduced when *veA* is deleted in another common *A. fumigatus* laboratory isolate, CEA10 (23). The basis for this discordance is not understood and, more broadly, highlights the potential for strain-to-strain variability concerning *A. fumigatus* developmental regulation. Indeed, more direct comparisons between Af293 and CEA10 have uncovered considerable differences at both the genomic and immunogenic levels (21, 24). The extent to which this reflects a greater heterogeneity across the species has been poorly explored to date.

In this report, we test the overt photoresponse of 15 *A. fumigatus* isolates to address the issue of strain heterogeneity on a larger scale. We show that while some isolates accumulate hyphal melanin as initially reported with Af293, most isolates induce conidiation similarly to *A. nidulans*. Through gene deletion analysis, we demonstrate that both responses are *lreA* dependent, further demonstrating that gene function and/or regulatory wiring can vary across isolates. Photobiological variability also existed with respect to germination kinetics and cell wall homeostasis, though not in a manner that correlated with the pigmentation or conidiation response. This unanticipated but extensive intraspecies heterogeneity in light responsiveness led us to examine virulence, and indeed virulence in a murine infection model also varied considerably across isolates and was also independent of the photoresponsive phenotype. Cumulatively, our data illuminate the significant and extensive biological and even pathogenic variability that can exist between isolates of *A. fumigatus* and likely other fungal species.

## RESULTS

### The overt photoresponse is variable across *A. fumigatus* isolates.

We predicted (i) that the genomic differences that exist between *A. fumigatus* strains Af293 and CEA10 reflect a greater heterogeneity within the species and (ii) that, as a result of this heterogeneity, the behavioral responses of different isolates to a common environmental stimulus would vary. To this end, we opted to use light as our behavioral cue since it can be easily applied and the photoresponse of Af293 is both well characterized and robust (20). Included in our analysis were three *A. fumigatus* isolates that serve as common “laboratory strains” and are well represented in the literature: Af293 (whose light response was previously assessed), CEA10, and H237 (25, 26). Also included were 12 uncharacterized isolates from a variety of clinical and environmental sources. A description of these strains can be found in Table S1 in the supplemental material.

**FIG 1:**
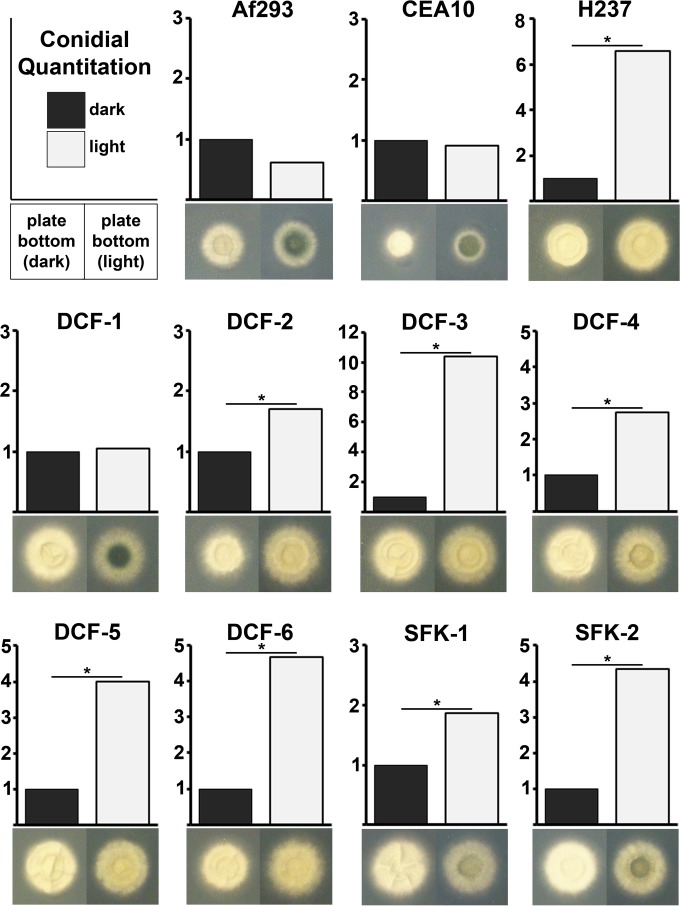
*A. fumigatus* isolates induce pigmentation or conidiation in response to light. Conidia of the indicated strain were point inoculated onto GMM (plate pictures) or RPMI 1640 (graphs) and incubated for 48 h either in constant darkness or under constant white light illumination. Conidiation values are normalized to the dark-grown sample of the indicated strain. Enumeration of conidia was performed in triplicate, and data were statistically analyzed by Student’s *t* test (*, *P* ≤ 0.05).

To test the effect of light on gross colony morphology, conidia were point inoculated onto glucose minimal medium (GMM) agar and incubated for 48 h at 37°C in the dark or under constant white light illumination. Consistent with our previous report, the major overt response of Af293 included the accumulation of a dark-green hyphal pigment observable on the colony reverse (plate bottom), presumably dihydroxy naphthalene (DHN) melanin (20, 27). This robust pigmentation was also observed in the CEA10 and DCF-1 isolates, and was relatively weaker in the SFK-1, SFK-2, and W72310 isolates. In the remaining 9 isolates (H237, DCF-2 to DCF-6, 47-4, 47-10, and 47-57), however, no appreciable accumulation of pigment was observed under either dark or light conditions (Fig. 1; see also Fig. S1 in the supplemental material). These data demonstrate that the photopigmentation response is not widely conserved across *A. fumigatus* isolates, though it is characteristic of the two most commonly utilized laboratory strains, Af293 and CEA10.

Light is known to induce asexual sporulation in a variety of fungi, including the models for fungal photobiology, *Aspergillus nidulans* (28) and *Neurospora crassa* (29). In contrast, we reported that light has a minor repressive role on conidiation in strain Af293 when grown on GMM (20). To determine if this is consistent across our various isolates, conidia were spread across GMM plates and incubated at 37°C in the dark or under constant white light. Following incubation, the conidia formed by the resulting mycelium were harvested and enumerated. Whereas the conidial counts for most isolates did not differ between dark and light environments, strains H237, DCF-4, and DCF-5 did display a slight increase under illumination (see Fig. S2A in the supplemental material).

Conidiation is strongly promoted on GMM, likely due to the inclusion of a reduced nitrogen source (ammonium). We therefore reasoned that an effect of light on development might be masked by the nutritional influence of the medium. Accordingly, we repeated the experiment on RPMI 1640 medium, on which conidiation is generally less robust (30). As with GMM, light had a minor repressive effect on conidiation for Af293 on RPMI following a 48-h incubation, indicating that the qualitative influences of light on the two media were similar. Light had no quantifiable effect on conidiation for the CEA10, DCF-1, 47-4, and W72310 isolates but did indeed lead to a statistically significant increase in conidial counts for all others (Fig. 1; see also Fig. S1 in the supplemental material). The degree of induction was variable among the strains, ranging from a less-than-2-fold induction for DCF-2 to a greater-than-10-fold induction for DCF-3.

Notably, the majority of *A. fumigatus* isolates tested here could be broadly categorized as “photopigmenters” (Af293, CEA10, DCF-1, and W72310) or “photoconidiators” (H237, DCF-2 to DCF-6, 47-10, and 47-57). In other words, strains appeared to induce their production either of hyphal pigments or of conidia in response to light but seldom both. Some exceptions included the SFK-1 and SFK-2 isolates, which displayed an induction of both processes, although the degree of pigmentation in the light was markedly weaker than that of Af293, CEA10, and DCF-1. Conversely, 47-4 failed to demonstrate any overt response to light (see Fig. S1 in the supplemental material). A summary of these overt photoresponses can be found in Table S2.

### Light-controlled conidial banding patterns are not observed under free-running conditions.

**FIG 2:**
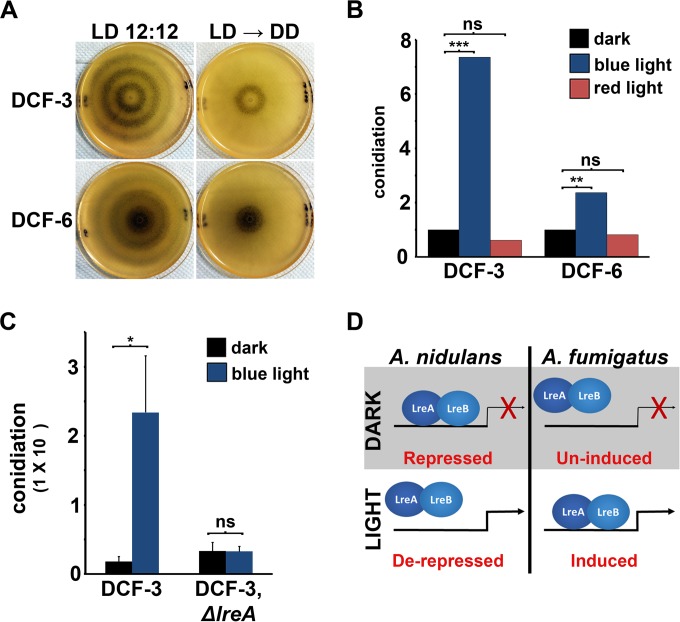
The photoconidiation response is blue light and *lreA* dependent. (A) Conidia were point inoculated onto RPMI 1640 plates and incubated for 6 days in an alternating 12-h-light/12-h-dark photocycle (left column). Alternatively, plates were transferred to constant darkness following an initial 2-day incubation in the 12-h-light/12-h-dark environment (right column). (B) Conidia were spread across RPMI 1640 plates and incubated for 48 h either in the dark or under constant illumination under red or blue LEDs. Conidial counts are normalized to the respective dark-grown sample. Experiments were performed in triplicate, and total conidia were compared by Student’s *t* test (**, *P* ≤ 0.01; ***, *P* ≤ 0.001; ns, not significant). (C) Conidia were spread across RPMI 1640 plates and incubated for 48 h either in constant darkness or under constant white light illumination. Experiments were performed in triplicate, and error bars represent the mean conidial counts (±standard deviation). Groups were compared by Student’s *t* test (*, *P* ≤ 0.05). (D) Scheme of LreA-dependent regulation of asexual development in *A. nidulans* versus *A. fumigatus*.

To test the effect of light on conidiation in a more natural photic environment, DCF-3 and DCF-6 were point inoculated onto RPMI plates and incubated in a 12-h-light/12-h-dark photocycle for 6 days. The colonies did not form conidia in the initial 24 h. Once developmental competence was reached, however, conidia were formed most prominently during the light phase of the photocycle, manifesting as conidial rings on the radially expanding colony (Fig. 2A). These data support a model in which subapical hyphal compartments are fixed as either differentiated or undifferentiated based on the photic environment encountered when those regions represented the growth front. Similar banding patterns of melanized and nonmelanized hyphae were previously observed with Af293 grown under identical photocycles (20).

Conidiation is driven both by light and by the circadian clock in *Neurospora crassa*, the result of the latter being a conidial banding pattern that persists when light-synchronized mycelia are transferred to constant darkness (31–33). Similarly, the periodic formation of sclerotia observed in light/dark cycles in *Aspergillus flavus* also persists after transfer to darkness (34). To determine if rhythmic conidiation would similarly manifest under constant (i.e., free-running) conditions in *A. fumigatus*, DCF-3 and DCF-6 were transferred to constant darkness following an initial 48 h of light/dark photocycles. After an initial band that corresponded to the second light cycle, no subsequent conidial rings were observed following the transfer to darkness (Fig. 2A). This suggests that for these two isolates, conidiation is tightly light regulated but not circadianly regulated under the conditions tested.

### The photoconidiation response is blue light and *lreA* dependent.

Conidiation is induced additively by blue and red light in *A. nidulans* (8). We hypothesized that this would be conserved in photoconidiating isolates of *A. fumigatus*, given that the fungus contains functional blue (LreA) and red (FphA) light receptors (20). To test this, the conidiation experiment on RPMI medium was repeated with DCF-3 and DCF-6 using red or blue light-emitting diodes (LEDs). For both isolates, only blue light induced conidiation beyond the levels found in darkness (Fig. 2B). Therefore, the wavelength dependency of the photoconidiation response differs between *A. fumigatus* and *A. nidulans*.

We further hypothesized a central role for LreA in regulating the photoconidiation response based upon the blue light dependency of the behavior. The DCF-3 isolate was selected as a representative of the photoconidiator subset for further analysis as it displayed the greatest degree of conidial induction by light (Fig. 1).

We first wanted to determine if the steady-state mRNA levels of *lreA* accumulate upon light exposure as had been reported in Af293 (20). To this end, DCF-3 was incubated in the dark for 48 h and then transferred to white light for 0, 15, 30, 60, or 120 min prior to RNA isolation. Reverse transcription-PCR (RT-PCR) and quantitative-reverse transcription PCR (qRT-PCR) analyses demonstrated a light-mediated increase of *lreA* transcript in this background (see Fig. S3A in the supplemental material; also data not shown), suggesting that *lreA* is similarly regulated in the two phenotypic subsets.

LreA of *A. nidulans* serves as a repressor of conidiation in the dark, the activity of which is released upon illumination (8, 11). We predicted that LreA would function in a similar way in *A. fumigatus* and, consequently, deletion of *lreA* would lead to increased conidiation in the dark (i.e., a derepression of asexual development). Accordingly, we replaced the *lreA* open reading frame with the hygromycin phosphotransferase (*hph*) gene in the DCF-3 background (see Fig. S3B in the supplemental material). Contrary to our prediction, conidiation of the *ΔlreA* mutant was unaffected in the dark; instead, the photoinduction of conidiation was completely lost in the mutant (Fig. 2C). This parallels what is observed in Af293, where the photopigmentation response is ablated upon *lreA* deletion (20). Moreover, this suggests that LreA serves as a light-induced activator of downstream processes (either pigmentation or conidiation) rather than as a repressor as in *A. nidulans*. A model reflecting this difference between the two aspergilli is proposed in Fig. 2D.

### The impact of light on conidial germination tracks independently of other photoresponses.

**FIG 3:**
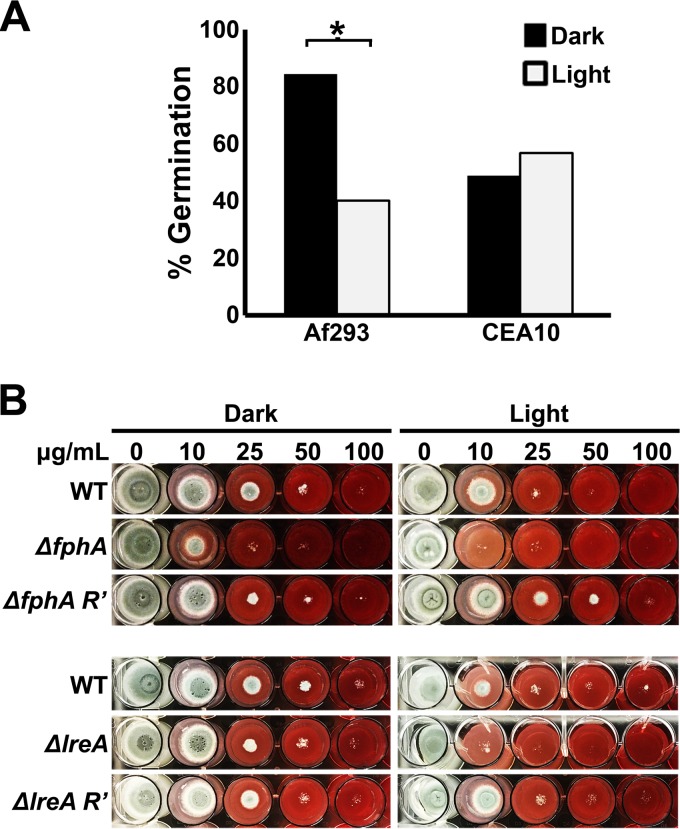
The photosensory control of germination and cell wall homeostasis differs between Af293 and CEA10. (A) Conidia were inoculated into GMM broth and incubated at 37°C for 8 h either in constant darkness or under constant illumination (blue plus red LEDs). A minimum of 100 conidia were scored for the presence or absence of a germ tube in each group, and light and dark samples were compared by the chi-square test (*, *P* ≤ 0.05). (B) Conidia of the indicated CEA10 genotype were spot inoculated onto GMM plus Congo red agar and incubated at 37°C for 48 h in constant darkness or under constant illumination (blue plus red LEDs).

Beyond photopigmentation, the other major effect of light on strain Af293 is the inhibition of germination kinetics (20). We postulated that all photoresponsive behaviors would cluster together among the isolates, and thus, the negative influence of light on germination would be observed only within the photopigmentation subset (e.g., Af293, CEA10, and DCF-1). To assess this, conidia were inoculated into GMM broth and incubated at 37°C under either constant dark or constant light conditions. Following 8 h of incubation, and contrary to our prediction, the proportion of germinated conidia was significantly reduced by light in eight of the 10 isolates tested (see Fig. S4 in the supplemental material). Illumination had no impact in this regard on CEA10 and DCF-4, the former of which was confirmed in subsequent experiments (Fig. 3A). These data indicate that specific photoresponses are variably distributed across *A. fumigatus* isolates in a seemingly unpredictable (i.e., unclustered) manner.

The germination response of Af293 is mediated by the phytochrome FphA (20). Therefore, we hypothesized that the inability of light to influence germination in CEA10 or DCF-4 was due to a nonfunctional *fphA* allele in those isolates. To gain some insight into this, the entire *fphA* coding sequence was sequenced from Af293, CEA10, DCF-3, DCF-4, and DCF-5. The only variation that existed across all sequences was observed in strain Af293, in which a single base pair substitution led to a glutamate-to-lysine missense mutation at position 988 (see Fig. S5 in the supplemental material). Because the phytochrome of Af293 is known to be functional, we reasoned that this mutation did not alter the function of the protein. Hence, the absence of a photorepressive effect on germination in some isolates remains mechanistically unclear.

### The influence of *lreA* on cell wall homeostasis differs between Af293 and CEA10.

Considering the above data, we wanted to further examine the extent to which specific photoreceptor functionality varied between isolates. For example, given that FphA apparently does not drive germination in CEA10, we wanted to know if it regulated any of the processes that it does drive in Af293. To this end, we deleted both *lreA* and *fphA* in the CEA10 background (see Fig. S3 in the supplemental material) and analyzed the phenotypes of the mutants with respect to several LreA- or FphA-dependent processes previously described (20).

To assess the phenotypes of the mutants with respect to the photopigmentation response, conidia were point inoculated onto GMM plates and incubated at 37°C either in constant darkness or under constant illumination for 72 h. Pigmentation of the mycelium persisted upon deletion of *fphA*, although the intensity of the response did appear somewhat attenuated in some of the independently isolated mutants (see Fig. S6 in the supplemental material). In contrast, photopigmentation was completely abolished in an *ΔlreA* mutant (see Fig. S6). These mutant phenotypes are similar to those observed for Af293, suggesting that the photoreceptor regulation of the pigmentation response is largely conserved.

Beyond driving germination in the dark in strain Af293, FphA also promotes cell wall homeostasis in the light (20). More completely, the fungus is hypersensitive to the cell wall-destabilizing drug Congo red in the light, and this sensitivity is exacerbated in the *ΔfphA* but not *ΔlreA* mutants. To determine if this was conserved in CEA10, conidia of the various mutants were incubated in the presence of Congo red for 48 h in constant darkness or under constant illumination. Similarly to Af293, the wild-type CEA10 was more sensitive to the dye under illumination, and this sensitivity was enhanced in the *ΔfphA* mutant (Fig. 3B). Surprisingly, the *ΔlreA* mutant was also more sensitive to Congo red in the light, indicating that LreA plays a more prominent role in regulating cell wall homeostasis in CEA10 than it does in Af293. Importantly, the mutant phenotypes were fully complemented upon reconstitution of the wild-type alleles (Fig. 3B). Cumulatively, these data indicate that the contribution of particular photoreceptors to a specific response varies to a surprising degree in a strain-dependent manner.

### Virulence is highly variable across isolates and does not correlate with the overt light response.

**FIG 4:**
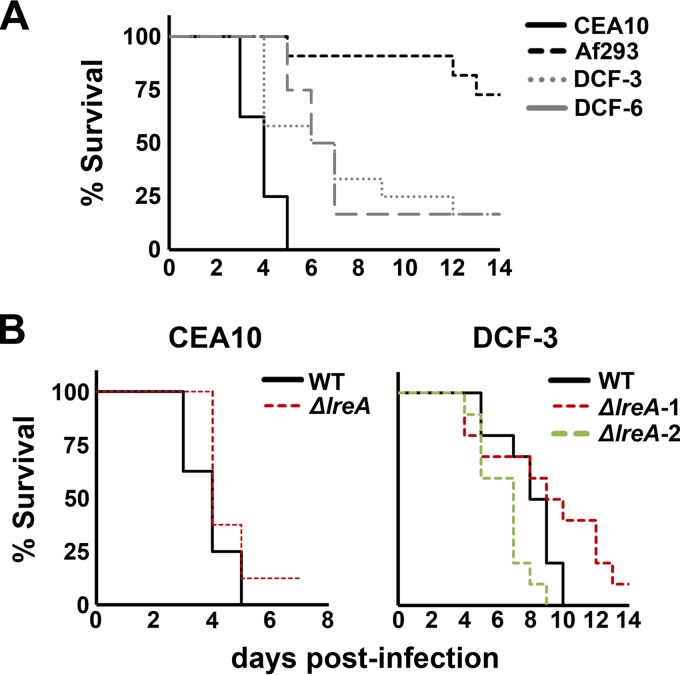
Virulence is variable across isolates and is independent of either the overt light response or *lreA*. (A) All animals were immunosuppressed with a single dose of triamcinolone acetonide (day −1) and inoculated intranasally with 1.0 × 10^6^ conidia of the indicated isolate (CEA10, *n* = 8; Af293, *n* = 11; DCF-3 and DCF-6, *n* = 12). (B) Left, CEA10 background. The wild-type curve is from the same experiment as that depicted in panel A; the *ΔlreA* group was run concurrently with the WT and under the same protocol. Survival curves are not statistically significant (*P* = 0.16). Right, DCF-3 background. Groups of 10 CD-1 mice were immunosuppressed with two doses of Kenalog (days −1 and +3) and inoculated intranasally with the indicated genotype. Two independently isolated *ΔlreA* mutants are shown. WT versus Δ*lreA*-1, *P* = 0.14; WT versus Δ*lreA*-2, *P* = 0.03. All statistical analyses were performed with the log rank test.

We postulated that the dichotomous nature of the overt light response (i.e., pigmentation versus conidiation) was reflective of a broader schism in how isolates interpret and/or respond to other environmental cues. Given that an appropriate response to certain stresses is an important pathogenicity determinant, e.g., the thermal stress (35) or hypoxic (36) response, we hypothesized that the virulence of a particular isolate would track with its overt photoresponse. To test this, we selected two photopigmenters (Af293 and CEA10) and two photoconidiators (DCF-3 and DCF-6) to compare in a murine model of invasive aspergillosis. Specifically, outbred mice were immunosuppressed with a single dose of triamcinolone acetonide (Kenalog-10) on the day preceding intranasal inoculation with 1.0 × 10^6^ fungal conidia. Cumulative mortality was then assessed for 2 weeks following inoculation. Consistent with our prediction, the survival curves associated with the DCF-3 and DCF-6 infected groups (photoconidiators) were statistically identical (Fig. 4A). However, the survival curves of CEA10 and Af293 (photopigmenters) were markedly distinct. All animals in the CEA10-infected group succumbed to infection within 5 days postinoculation, providing a curve that is statistically different from the DCF-3 and DCF-6 curves (*P* = 0.004, log rank test). In contrast, the majority of animals infected with Af293 survived for the entire 2-week duration, the curve for which was also statistically distinct from the others (Fig. 4A). Thus, the virulence of *A. fumigatus* isolates is strikingly variable, although not in a way that appears to correlate with their overt light responses.

### Loss of *lreA* does not reduce virulence in a photopigmenter or photoconidiator background.

Deletion of the *lreA* ortholog in the basidiomycete yeast *Cryptococcus neoformans* and the ascomycete mold *Fusarium oxysporum* leads to a loss of virulence in their respective mouse models of infection (6, 7). To assess whether the contribution of LreA to virulence is conserved in *A. fumigatus*, the *ΔlreA* mutants in the CEA10, Af293, and DCF-3 backgrounds were also analyzed in either the same experiment described for Fig. 4A or one similar.

For the CEA10 background, two independently derived mutants were tested alongside the wild type (WT) in the experiment depicted in Fig. 4A. The wild-type curve was simply replotted in Fig. 4B so that a direct comparison with the *ΔlreA* curves could be visualized. Moreover, one of the mutant curves was discarded as the two isolates yielded identical results. The wild-type and *ΔlreA* curves were statistically indistinguishable, indicating that loss of *lreA* does not attenuate virulence in the CEA10 background.

The DCF-3 wild type was compared directly with two of its *ΔlreA* isolates in a study separate from that shown in Fig. 4A. In this experiment (Fig. 4B), all animals still appeared healthy at day +3; therefore, an additional injection of Kenalog was administered on that day in order to facilitate more robust mortality curves and, consequently, our ability to distinguish a difference between the genotypes. Both of the tested mutants yielded curves that resembled that of the wild type, though slight differences are noticeable: the *ΔlreA*-1 strain appears to be slightly right shifted, but it is not statistically different from the wild type (*P* = 0.14; log rank test); the *ΔlreA*-2 strain, on the other hand, is slightly left shifted (*P* = 0.025). As the mutant curves are clustered closely to either side of the wild type, we concluded that *lreA* does not attenuate virulence in this photoconidiating background.

The Af293 strains were tested in experiments independent of that described for Fig. 4A. First, the wild type and *ΔlreA* mutant were tested in a single-dose Kenalog model (day −1) with an inoculum of 2.0 × 10^6^ conidia. Second, the wild type and the *ΔlreA ΔfphA* mutant, generated previously (20), were tested in both a multidose Kenalog model (days −1 and −3; for the same reasons as those outlined for the DCF-3 background) and a chemotherapy (neutropenia) model involving an administration of both Kenalog and cyclophosphamide. In all cases, the mutants were indistinguishable from their respective wild-type controls (see Fig. S7 in the supplemental material). Taken together then, our results suggest that loss of *lreA* does not attenuate virulence in *A. fumigatus* in any of the photoresponsive subsets.

## DISCUSSION

Upon our initial assessment of the *A. fumigatus* photoresponse (20), we concluded that light’s major influence on the mycelium is to induce pigmentation, rather than asexual development. Although that study was limited to only a single isolate, the commonly used Af293 strain, we predicted that the behavior would be conserved across the species; we have formally tested this here through the analysis of 15 environmental and clinical isolates. Much to our surprise, we found that other, indeed most, *A. fumigatus* isolates do display a marked induction of sporulation in the light. Moreover, these “photoconidiators” typically fail to display the light induction of pigmentation observed in Af293. Thus, completely different behaviors manifest across isolates in response to a common signal. We believe that there are two important conclusions that emerge from these data: the proximate one, pertaining to the photobiology of *A. fumigatus*, and the description of intraspecies heterogeneity, which has a broader significance with respect to how we understand the biology of *A. fumigatus* and other fungi. As these points are interwoven, so too will be our treatment of them here.

At the interspecies level, the photoconidiation phenotype of some *A. fumigatus* isolates appears to resemble that of *A. nidulans*, i.e., conidial production is more robust upon illumination. However, there are key behavioral and genetic differences between the two aspergilli in this regard. First, whereas both blue and red light additively induce conidiation in *A. nidulans* (8), we have observed that only blue light has a sporogenic effect in *A. fumigatus*. Second, the exact role of LreA in regulating conidiation appears to be different between the two species. In *A. nidulans*, LreA acts as a repressor of conidiation as evidenced by the fact that *ΔlreA* mutants display a slight increase in conidiation in both the dark and the light (8, 9). In contrast, we have observed that the DCF-3 *ΔlreA* mutant displays wild-type (WT) levels of conidiation in the dark but fails to induce conidiation in the light. From these data, we infer that LreA in *A. fumigatus* serves primarily as an inducer of conidiation in the light, with little or no developmental influence in the dark (Fig. 2D). In this way, *lreA* of *A. fumigatus* more closely mimics *wc-1* of *N. crassa*, the deletion of which leads to an ablation of light-induced processes, including conidiation. Last, the cooperative interaction between LreA and FphA seen in *A. nidulans* is apparently not conserved in *A. fumigatus* with respect to conidiation. In *A. nidulans*, the photoinduction of conidiation is lost only upon deletion of both *lreA* and the phytochrome gene, *fphA* (8). However, deletion of *lreA* was sufficient to ablate the conidiation response in the *A. fumigatus* DCF-3 background. Taken together, we conclude that the regulation of the photomorphogenic response is divergent between *A. fumigatus* and *A. nidulans*. Given the nature of our findings, however, we realize that the analysis of additional isolates may reveal that the distinctions that we have just outlined also occur within either species.

Our assessment of basic growth parameters, i.e., independent of the light environment, revealed continuous (quantitative) variability across our isolates; this included baseline conidiation levels, germination, and linear growth rates (see Fig. S2 and S4 in the supplemental material). This data set then adds to a small number of studies that, in addition to growth, also report variation in antifungal susceptibility across *A. fumigatus* isolates (37–39). Of course, we also identified quantitative variability with respect to the light response. For example, the degree to which spore production was induced by light in our photoconidiators ranged between 2- and 10-fold. The extents to which light reduced germination or induced pigmentation (in the photopigmenting subset) were also variable. There are a limited number of studies that suggest that such photobiological variability may be common among fungi. Across 69 isolates of *N. crassa*, for example, the induction of mycelial pigments by light ranged continuously between 5- and 70-fold (average, 26-fold). That range of induction was similarly variable in *Neurospora discreta* (2.9- to 31.9-fold; *n* = 63) and *Neurospora tetrasperma* (9.8- to 41.7-fold; *n* = 17). Interestingly, the degree of photocarotenogenesis in each of these species was inversely correlated with the latitude at which the isolate was collected, indicating that light/UV intensity is a selective pressure on this parameter (40). Related to light sensing in *N. crassa* is the circadian clock, for which WC-1 (LreA in *Aspergillus*) is a central player in both. An analysis of 118 isolates revealed that circadian period varied between 18 and 24 h (average, 22 h) and that phase varied between 5 and 11 h (average, 7 h). As with photocarotenogenesis, the period remarkably correlated with latitude of isolation. The period length was furthermore attributed to the variation in the *wc-1* gene itself, namely, the length of the N-terminal poly(Q) region (41). Such a poly(Q) region does not exist in the LreA protein of the aspergilli. The general conclusion from these data is that while there are significant differences in the degree to which light affects processes, there are very few instances in *Neurospora* in which light is without an effect or has a reverse effect.

The gross/colonial impact of light on *A. fumigatus* is also qualitatively divergent across isolates, manifesting as either stress resistant (pigmentation) or developmental (conidiation) in nature. We appreciate that the distinction between a “stress” and “development” response in this way may be an oversimplification, as sporulation itself may be induced under stress conditions, e.g., nutrient stress (30). Nevertheless, most isolates do appear to divert resources to one response or the other in light but not to both. Such a dichotomy with respect to light and development has also been noted in the plant pathogen *Botrytis cinerea*. Most field isolates of this mold preferentially form conidiophores (asexual) in the light but sclerotia (sexual development) in the dark (42, 43). Some isolates, on the other hand, are defective in sclerotium development and instead robustly produce conidia even in the dark. At face value, this appears to represent a variance in the light response comparable to what we describe here for *A. fumigatus*, i.e., isolates are split based on whether or not light can influence development. However, the “always-conidia” phenotype in *B. cinerea* is attributed to a single nucleotide polymorphism (SNP) in *bcvel1*, the ortholog to *A. nidulans veA*, which leads to a premature truncation (44). In this way, the *bcvel1* mutants are comparable to the *veA1* mutant of *A. nidulans* in that both are locked in an asexual developmental state. Thus, this would be better described as allelic variation of *bcvel1* rather than photobiological variation in the way that we describe for *A. fumigatus*. First, sequence analysis of the *veA*-coding sequence from eight of our isolates revealed only one point mutation, leading to a threonine-to-isoleucine missense, that was common to both DCF-1 (a photopigmenter) and DCF-5 (a photoconidiator) (see Fig. S8 in the supplemental material). Therefore, alteration in the VeA protein structure does not appear to be the basis for the *A. fumigatus* phenotypic variation. Second, the *A. fumigatus* strains that are developmentally insensitive to light are still photoresponsive in other ways, i.e., they induce mycelial pigments. It is currently unclear then what the molecular/genetic basis of the variation is, but it appears to involve a fundamental rewiring of the light-responsive machinery in some isolates, i.e., light drives one response or another. Comparison of the Af293 and CEA10 genomes demonstrates that considerable variation can exist between the two. For example, each strain harbors genes not found in the other; as for Af293, these lineage-specific genes may be involved in arsenic and betaine metabolism as well as osmotic and heavy metal stress (21). As neither of the available *A. fumigatus* genomes is derived from photoconidiators, however, genomic variances that could account for the altered light responses are unknown. Future studies will be aimed at elucidating such genomic, genetic, or epigenetic differences. In any case, and to our knowledge, these data are the first describing qualitative variations with respect to a fungal light response.

We suggest that the opposing photoresponses of *A. fumigatus* are a bellwether for other qualitative divergences in this and other species. Reports of this sort are sparse, however, inasmuch as the phenomenon has been poorly investigated. One exception comes from the host-pathogen interaction, where it has been shown that different laboratory strains can elicit qualitatively distinct immune responses. Specifically, strain Af293 induces a robust proinflammatory reaction in dendritic and mononuclear cells (measured by the release of tumor necrosis factor alpha [TNF-α] and gamma interferon [IFN-γ]); in contrast, CEA10 and Af300, another common laboratory strain, both elicit a hyperinflammatory response (marked by interleukin 17A [IL-17A] induction) (24). Assuming that the degree of genomic variation observed between Af293 and CEA10 can be applied across other isolates, diverged behaviors or characteristics will likely reveal themselves upon comprehensive/multi-isolate investigations.

To the extent that overt behaviors can be diverged, so too must the functionality of the related proteins. In our work, this is most obvious with respect to LreA driving either a pigmentation or a conidiation response upon illumination. More subtle was our investigation into LreA’s involvement in regulating cell wall homeostasis. Whereas deletion of *lreA* leads to a hypersensitivity to Congo red in CEA10, the mutant has no phenotype in this regard in Af293 (20). Therefore, our understanding of how or whether LreA contributes to cell wall homeostasis is fundamentally altered as we change the wild-type background. Similar findings appear in the literature only anecdotally. For example, phenotypes associated with *veA* deletion (described in the introduction) are completely opposite in Af293 and CEA10, but these findings came from separate publications in separate groups (22, 23). In another example, the deletion of the transcription factor *crzA* in Af293 gives rise to a mutant with a severely stunted radial growth phenotype (45); alternatively, the same mutant in the CEA10 background displays a growth rate identical to that of the wild type under standard growth conditions (46). Therefore, conclusions about protein functionality, as with overt behavior, should be considered on a strain-to-strain basis.

Several lines of evidence from other fungi support a connection between photosensory pathways and fungal pathogenesis in mammalian hosts. First, mice infected with *Histoplasma capsulatum* yeasts that were cultured in light lived longer than those infected with yeasts cultured in constant darkness. Moreover, this influence of light was observed for one common serotype of *H. capsulatum*, but no effect was observed in another serotype (47). Thus, not only can light-sensing pathways augment fungal virulence, but they can do so in a strain-dependent manner. This warranted our investigation into whether the virulence of *A. fumigatus* could be correlated with the photobiological response (i.e., photoconidiators versus photopigmenters). Although we could not correlate survival curves of *A. fumigatus* with a photobiological phenotype, we did observe considerable variability in a more general way. As with the abovementioned study using immunocompetent mice (24), CEA10 was associated with the highest mortality in our immunocompromised model. More completely, CEA10 was statistically more virulent than two photoconidiators, which were in turn statistically more virulent than Af293. Anecdotally, strain differences with respect to *A. fumigatus* virulence have been discussed for some time; to our knowledge, however, this is the first report in which it has been formally tested, i.e., different strains under identical experimental parameters, such as inoculum size, immunosuppressive regimen, etc.

The only consistent aspect of our infection studies, and indeed at any phenotypic level, was that deletion of *lreA* did not lead to an appreciable attenuation of virulence in either a photopigmenting (CEA10 and Af293) or photoconidiating (DCF-3) background of *A. fumigatus*. This is in contrast to both *C. neoformans* and *F. oxysporum*, in which deletion of *lreA/wc-1* was associated with reduced mortality in a murine model (6, 7). How a blue light photoreceptor might contribute to pathogenesis is currently unclear in both cases, but the data nevertheless suggest that photoperception and virulence may be linked in other human fungal pathogens. This is a particularly attractive prospect for fungal infections in which the light environment can be readily manipulated, for example, ocular fungal infections (keratitis) caused by *Fusarium* species. For our part, ongoing investigations into how photoreceptors influence *A. fumigatus* metabolism and stress resistance may ultimately reveal a role for these proteins in regulating pathogenic potential.

In conclusion, our work, in combination with the growing literature, strongly indicates that genetic background must carefully be considered when making conclusions about behavioral responses or gene functionality. Our initial characterization of Af293, for instance, led us to conclude that light fundamentally regulates development differently in *A. fumigatus* and *A. nidulans* (20). The inclusion of additional isolates in the former has now significantly changed that interpretation. Thus, results derived from studies involving a single strain may not be applicable to the species as a whole and conclusions concerning species-to-species divergence should be reserved.

## MATERIALS AND METHODS

### *Aspergillus fumigatus* strains utilized for this study.

Deletion of the *lreA* and *fphA* genes from the CEA10 and DCF-3 backgrounds was performed as described previously (20). Briefly, fungal protoplasts were transformed with two constructs designed to replace the entire coding sequence with either the hygromycin phosphotransferase (*hph*) or phleomycin resistance (*bleR*) gene. The deletion of the respective loci in drug-resistant transformants was initially determined by genomic PCR screening and confirmed by RT-PCR analysis as previously described (20) and depicted in Fig. S3 in the supplemental material.

### RNA isolation and RT-PCR analysis.

Following culturing as described above, mycelial tissue was harvested by brief vacuum filtration followed by freezing in liquid nitrogen. Total RNA was isolated from the frozen tissue by Trizol extraction with the aid of a TissueLyser (Qiagen). Eight hundred nanograms of RNA was used for first-strand cDNA synthesis (Superscript III; Invitrogen). For quantitative RT-PCR analysis, a 1:2 or 1:10 dilution of cDNA was used in a 20-µl PCR mixture using Phusion Taq polymerase (New England Biolabs) and actin (*actA*) or *lreA* primers described previously (20).

### Growth conditions and light treatment.

All conidial inocula were harvested from glucose minimal medium (GMM) plates containing 1% glucose and ammonium tartrate as a nitrogen source, as described in reference 48. RPMI 1640 agar contained, per liter, 10.4 g RPMI 1640 powder (Sigma; R6504-10L), 34.53 g morpholinepropanesulfonic acid (MOPS), 18 g glucose, and 15 g Bacto agar; pH was adjusted to 6.9 to 7.1. YG broth contained 2% glucose and 0.5% yeast extract.

All incubators were placed in a dark room, and “dark” samples were isolated with all lights off with the aid of a near-infrared imaging goggle. For white light-treated cultures, plates or flasks (as indicated) were irradiated under cool fluorescent light bulbs (General Electric F20T12-CW) emitting light over a broad spectrum from 400 to 700 nm; total light intensity was ~40 µmol/photon/m^2^/s. For blue and red light treatments, plates were incubated in an E-30LED growth chamber equipped with blue and red light-emitting diodes (Percival Scientific, Inc., Perry, IA). All incubations were performed at 37°C.

### Quantitation of conidiation.

To quantify total conidia produced by the various strains, 300 µl of a 1.0 × 10^6^/ml conidial suspension was spread across a 100-mm RPMI 1640 agar plate and incubated at 37°C under either constant dark or white light conditions as described above. After 48 h of incubation, conidia were harvested by swabbing conidia into 15 ml of sterile water three times (45 ml total). Dilutions of the conidial suspensions were performed in triplicate, and conidia were enumerated with a hemacytometer. Conidial counts from the dark-grown and illuminated groups were compared with Student’s *t* test.

To compare the inducing effects of blue and red light, 120 µl of a 1.0 × 10^6^/ml conidial suspension was spread plated across 60-mm RPMI 1640 plates and incubated under constant dark, blue light, or red light conditions at 37°C for 48 h. Conidia were harvested by swabbing conidia into 8 ml of sterile water three times. Three plates were used per group.

### Germination assays.

Conidia of the various strains were inoculated in GMM broth at a density of 2.0 × 10^5^/ml and incubated at 37°C under the indicated light conditions. Following an 8-h incubation, a minimum of 100 conidia were scored for the presence or absence of a germ tube using a bright-field microscope. The statistical differences between groups were determined pairwise with the chi-square test.

### Sequencing of *veA* and *fphA.*

The entire coding sequence of *veA* (AFUA_1G12490) was amplified from each of the strains using Phusion Taq (Qiagen) and the following primers: forward, 5′-ACCTCCGTGATTTCTGGCT, and reverse, 5′-GAACATAATCTTTCCACTGGC. The entire 2-kb PCR product was sequenced from both ends at the Molecular Biology Core at Dartmouth using the following primers: forward 1, 5′-ACCTCCGTGATTTCTGGCT; forward 2, 5′-ACATCAAGGATGCGGACAAG; forward 3, 5′-ACCGTCCAAGCTATTCCAAG; reverse 1, 5′-GAACATAATCTTTCCACTGGC; reverse 2, 5-AAGTGGCGTCGTACTCGGTG; and reverse 3, 5′-CGTACAAGTGGAAGCTCAAC.

The entire coding sequence of *fphA* was amplified with the following primers: forward, 5′-TCTTGGCCTCAAATCTGTGG, and reverse, 5′-AATAGAGCAGAGAAAAAGC. The entire 5.7-kb PCR product was sequenced from both ends at the Molecular Biology Core at Dartmouth using the following primers: forward 1, 5′-ATGATGGTATAGCCACGTCG; forward 2, 5′ CAATCCCTTGAGTGTTTCCG; forward 3, 5′-CACTAAAGACTTTCCCGACC; forward 4, 5′-GGCCAGTTGAGTGTTCGATC; reverse 1, 5′-AGAGAAAAAGCTGTAGGAGG; reverse 2, 5′-ATTCGGATCGCTGTCATTGG; reverse 3, 5′-TGGTCAGATCCAGAAGGTCG; reverse 4, 5′-GTCATGTCTACAGGCGTTTG; and reverse 5, 5′-TGTGGATCGGTTCGTCTTCG.

### Congo red sensitivity assays.

Sensitivity to Congo red was tested as previously described (20). Briefly, GMM agar was supplemented with various concentrations of Congo red after autoclaving of the medium. All incubations were performed at 37°C for 48 to 72 h in the indicated light environment.

### Virulence assays.

In all experiments, groups of female, CD-1 mice, 19 to 21 g, were obtained from Charles River Laboratories. At a minimum, animals were injected subcutaneously with 40 mg/kg of body weight of triamcinolone acetonide (Kenalog-10; Bristol-Myers Squibb) on the day preceding fungal inoculation (day −1). On day 0, animals were anesthetized in an isoflurane chamber and inoculated intranasally with fungal conidia resuspended in 30 µl of phosphate-buffered saline (PBS). Conidial densities of the inocula varied between experiments and are specified in the figure legends. Moreover, the animals in the DCF-3 experiment (Fig. 4B) as well as the Af293 experiment shown in Fig. S7B in the supplemental material received a second 40-mg/kg injection on day +3. For the chemotherapy model (see Fig. S7C), animals received both the 40-mg/kg Kenalog injection on day −1 and a 150-mg/kg intraperitoneal injection of cyclophosphamide (Baxter Health Care Corporation) on days −1 and +3.

In all cases, moribund animals were euthanized by CO_2_ asphyxiation. Mortality curves between groups were compared by the log rank test.

## SUPPLEMENTAL MATERIAL

Figure S1  Photopigmentation and photoconidiation responses of additional *A. fumigatus* isolates. Conidia of the indicated strain were point inoculated onto GMM (plate pictures) or RPMI 1640 (graphs) and incubated for 48 h either in constant darkness or under constant white light illumination. Conidiation values are normalized to the dark-grown sample of the indicated strain. Enumeration of conidia was performed in triplicate, and data were statistically analyzed by Student’s *t* test (*, *P* ≤ 0.05). Download Figure S1, TIF file, 50.2 MB

Figure S2  (A) The photoconidiation response is more pronounced on RPMI 1640 medium (R) than on GMM (G). Experiments were performed in triplicate, and groups were compared by Student’s *t* test (*, *P* ≤ 0.05). (B) The overall conidiation levels are variable across the strains. Values are normalized to the Af293 DD sample. (C) The effect of light on growth rate is minimal for each of the tested isolates. Conidia were point inoculated on GMM and incubated for 3 days at 37°C in constant darkness or under constant white light illumination. Download Figure S2, TIF file, 66.9 MB

Figure S3  (A) RT-PCR analysis of *lreA* expression. Strains were incubated in the dark for 48 h and then transferred to constant white light illumination for the indicated time points. (B) Schematic depiction of the split-marker deletion strategy of *lreA* is shown as well as an RT-PCR demonstrating loss of the *lreA* transcript in the putative knockouts. *hph*, hygromycin phosphotransferase gene. (C) Schematic depiction of the split-marker deletion strategy of *fphA* is shown as well as an RT-PCR demonstrating loss of the *fphA* transcript in either the CEA10 WT or *ΔlreA* background. (D) RT-PCR demonstrating the transcript of either *lreA* (top) or *fphA* (bottom) in the respective complemented strain. Download Figure S3, TIF file, 40.8 MB

Figure S4  Light decreases germination rates in most *A. fumigatus* isolates. Conidia were inoculated into GMM and incubated for 8 h either in constant darkness or under constant white light illumination. A minimum of 300 conidia were scored for the presence or absence of a germ tube in each group, and light and dark samples were compared by the chi-square test (*, *P* ≤ 0.05). Download Figure S4, TIF file, 9 MB

Figure S5  FphA protein sequence alignment based on Sanger sequencing from the indicated isolates. Download Figure S5, TIF file, 52.1 MB

Figure S6  Additional phenotypes of the CEA10 photoreceptor mutants. (A) Pigmentation. Conidia were point inoculated into GMM plates and incubated at 37°C for 48 h either in constant darkness or under constant illumination (blue plus red LEDs). Several independent isolates of the *ΔfphA* mutation are shown and are distinguished numerically (e.g., 5-1, 7-2). (B) Congo red sensitivity. Conidia were point inoculated onto GMM plates supplemented with the indicated concentration of Congo red. Plates were then incubated for 48 h at 37°C for 48 h either in constant darkness or under constant illumination (blue plus red LEDs). Download Figure S6, TIF file, 26.5 MB

Figure S7  Deletion of *lreA* in the Af293 background does not attenuate virulence. (A) Single-dose steroid model. Groups of 12 CD-1 mice were immunosuppressed with Kenalog-10 (day −1) and inoculated intranasally with 2.0 × 10^6^ conidia of the indicated genotype. (B) Multidose steroid model. Groups of 16 CD-1 mice were immunosuppressed with two doses of Kenalog-10 (days −1 and +3) and inoculated intranasally with 1.5 × 10^6^ conidia of the indicated genotypes. Survival curves are not statistically different (*P* = 0.22, log rank test). (C) Chemotherapy (neutropenia model). Groups of 17 CD-1 mice were immunosuppressed with cyclophosphamide (days −2 and +3) and Kenalog-10 (day −1) and inoculated intranasally with 2.0 × 10^6^ conidia of the indicated genotypes. Survival curves are not statistically different (*P* = 0.4, log rank test). Download Figure S7, TIF file, 32.4 MB

Figure S8  VeA protein alignment based on Sanger sequencing from the indicated isolates. Download Figure S8, TIF file, 63.3 MB

Table S1  Strains used in this study Table S1, DOCX file, 0.1 MB

Table S2  Summary of photoresponsive behaviors in the analyzed isolates. Photopigmenting isolates are shaded. Table S2, DOCX file, 0.1 MB

## References

[1] Rodriguez-Romero J, Hedtke M, Kastner C, Müller S, Fischer R 2010 Fungi, hidden in soil or up in the air: light makes a difference. Annu Rev Microbiol 64:585–610. doi:10.1146/annurev.micro.112408.134000. 20533875

[2] Idnurm A, Verma S, Corrochano LM 2010 A glimpse into the basis of vision in the kingdom Mycota. Fungal Genet Biol 47:881–892. doi:10.1016/j.fgb.2010.04.009. 20451644 PMC2950209

[3] Fuller KK, Loros JJ, Dunlap JC 2015 Fungal photobiology: visible light as a signal for stress, space and time. Curr Genet 61:275–288. doi:10.1007/s00294-014-0451-0. 25323429 PMC4401583

[4] Froehlich AC, Liu Y, Loros JJ, Dunlap JC 2002 White collar-1, a circadian blue light photoreceptor, binding to the frequency promoter. Science 297:815–819. doi:10.1126/science.1073681. 12098706

[5] He Q, Cheng P, Yang Y, Wang L, Gardner KH, Liu Y 2002 White collar-1, a DNA binding transcription factor and a light sensor. Science 297:840–843. doi:10.1126/science.1072795. 12098705

[6] Idnurm A, Heitman J 2005 Light controls growth and development via a conserved pathway in the fungal kingdom. PLoS Biol 3:e95. doi:10.1371/journal.pbio.0030095. 15760278 PMC1064852

[7] Ruiz-Roldán MC, Garre V, Guarro J, Mariné M, Roncero MI 2008 Role of the White Collar 1 photoreceptor in carotenogenesis, UV resistance, hydrophobicity, and virulence of *Fusarium oxysporum*. Eukaryot Cell 7:1227–1230. doi:10.1128/ec.00072-08. 18503005 PMC2446679

[8] Purschwitz J, Müller S, Kastner C, Schöser M, Haas H, Espeso EA, Atoui A, Calvo AM, Fischer R 2008 Functional and physical interaction of blue- and red-light sensors in *Aspergillus nidulans*. Curr Biol 18:255–259. doi:10.1016/j.cub.2008.01.061. 18291652

[9] Bayram O, Braus GH, Fischer R, Rodriguez-Romero J 2010 Spotlight on *Aspergillus nidulans* photosensory systems. Fungal Genet Biol 47:900–908. doi:10.1016/j.fgb.2010.05.008. 20573560

[10] Ballario P, Vittorioso P, Magrelli A, Talora C, Cabibbo A, Macino G 1996 White collar-1, a central regulator of blue light responses in *Neurospora*, is a zinc finger protein. EMBO J 15:1650–1657. 8612589 PMC450076

[11] Hedtke M, Rauscher S, Röhrig J, Rodríguez-Romero J, Yu Z, Fischer R 2015 Light-dependent gene activation in Aspergillus nidulans is strictly dependent on phytochrome and involves the interplay of phytochrome and white collar-regulated histone H3 acetylation. Mol Microbiol 97:733–745. doi:10.1111/mmi.13062. 25980340

[12] Blumenstein A, Vienken K, Tasler R, Purschwitz J, Veith D, Frankenberg-Dinkel N, Fischer R 2005 The *Aspergillus nidulans* phytochrome FphA represses sexual development in red light. Curr Biol 15:1833–1838. doi:10.1016/j.cub.2005.08.061. 16243030

[13] Brandt S, von Stetten D, Günther M, Hildebrandt P, Frankenberg-Dinkel N 2008 The fungal phytochrome FphA from *Aspergillus nidulans*. J Biol Chem 283:34605–34614. doi:10.1074/jbc.M805506200. 18931394 PMC3259886

[14] Yu Z, Armant O, Fischer R 2016 Fungi use the SakA (HogA) pathways for phytochrome-dependent light signaling. Nat Microbiol 1:16019. doi:10.1038/nmicrobiol.2016.19. 27572639

[15] Purschwitz J, Müller S, Fischer R 2009 Mapping the interaction sites of *Aspergillus nidulans* phytochrome FphA with the global regulator VeA and the white collar protein LreB. Mol Genet Genomics 281:35–42. doi:10.1007/s00438-008-0390-x. 18936976

[16] Kafer E 1965 The origin of translocations in *Aspergillus nidulans*. J Genet 46:1583–1609. 10.1093/genetics/52.1.217 PMC1210839 5857597

[17] Mooney JL, Yager LN 1990 Light is required for conidiation in *Aspergillus nidulans*. Genes Dev 4:1473–1482. doi:10.1101/gad.4.9.1473. 2253875

[18] Bayram O, Krappmann S, Ni M, Bok JW, Helmstaedt K, Valerius O, Braus-Stromeyer S, Kwon NJ, Keller NP, Yu JH, Braus GH 2008 VelB/VeA/LaeA complex coordinates light signal with fungal development and secondary metabolism. Science 320:1504–1506. doi:10.1126/science.1155888. 18556559

[19] Sarikaya Bayram O, Bayram O, Valerius O, Park HS, Irniger S, Gerke J, Ni M, Han KH, Yu JH, Braus GH 2010 LaeA control of velvet family regulatory proteins for light-dependent development and fungal cell-type specificity. PLoS Genet 6:e1001226. doi:10.1371/journal.pgen.1001226. 21152013 PMC2996326

[20] Fuller KK, Ringelberg CS, Loros JJ, Dunlap JC 2013 The fungal pathogen Aspergillus fumigatus regulates growth, metabolism, and stress resistance in response to light. mBio 4:e00142-13. doi:10.1128/mBio.00142-13. 23532976 PMC3604765

[21] Fedorova ND, Khaldi N, Joardar VS, Maiti R, Amedeo P, Anderson MJ, Crabtree J, Silva JC, Badger JH, Albarraq A, Angiuoli S, Bussey H, Bowyer P, Cotty PJ, Dyer PS, Egan A, Galens K, Fraser-Liggett CM, Haas BJ, Inman JM, Kent R, Lemieux S, Malavazi I, Orvis J, Roemer T, Ronning C, Sundaram J, Sutton F, Turner G, Venter J, White O, Whitty B, Youngman P, Wolfe K, Goldman G, Wortman J, Jiang B, Denning D, Nierman W 2008 Genomic islands in the pathogenic filamentous fungus *Aspergillus fumigatus*. PLoS Genet 11:e1000046. doi:10.1371/journal.pgen.1000046. 18404212 PMC2289846

[22] Park H, Bayram Ö, Braus GH, Kim SC, Yu J 2012 Characterization of the velvet regulators in *Aspergillus fumigatus*. Mol Microbiol 86:937–953. doi:10.1111/mmi.12032. 22970834

[23] Dhingra S, Andes D, Calvo AM 2012 VeA regulates conidiation, gliotoxin production, and protease activity in the opportunistic human pathogen *Aspergillus fumigatus*. Eukaryot Cell 11:1531–1543. doi:10.1128/EC.00222-12. 23087369 PMC3536283

[24] Rizzetto L, Giovannini G, Bromley M, Bowyer P, Romani L, Cavalieri D 2013 Strain dependent variation of immune responses to *A. fumigatus*: definition of pathogenic species. PLoS One 8:e56651. doi:10.1371/journal.pone.0056651. 23441211 PMC3575482

[25] Fortwendel JR, Fuller KK, Stephens TJ, Bacon WC, Askew DS, Rhodes JC 2008 *Aspergillus fumigatus* RasA regulates asexual development and cell wall integrity. Eukaryot Cell 7:1530–1539. doi:10.1128/EC.00080-08. 18606827 PMC2547073

[26] Lopes Bezerra LM, Filler SG 2004 Interactions of *Aspergillus fumigatus* with endothelial cells: internalization, injury, and stimulation of tissue factor activity. Blood 103:2143–2149. doi:10.1182/blood-2003-06-2186. 14630829

[27] Langfelder K, Streibel M, Jahn B, Haase G, Brakhage AA 2003 Biosynthesis of fungal melanins and their importance for human pathogenic fungi. Fungal Genet Biol 38:143–158. doi:10.1016/S1087-1845(02)00526-1. 12620252

[28] Ruger-Herreros C, Rodríguez-Romero J, Fernández-Barranco R, Olmedo M, Fischer R, Corrochano LM, Canovas D 2011 Regulation of conidiation by light in *Aspergillus nidulans*. Genetics 188:809–822. doi:10.1534/genetics.111.130096. 21624998 PMC3176103

[29] Lauter F, Yamashiro CT, Yanofsky C 1997 Light stimulation of conidiation in *Neurospora crassa*: studies with the wild-type strain and mutants *Wc-1*, *Wc-2* and *acon*-*2*. J Photochem Photobiol B 37:203–211. doi:10.1016/S1011-1344(96)07405-2.

[30] Twumasi-Boateng K, Yu Y, Chen D, Gravelat FN, Nierman WC, Sheppard DC 2009 Transcriptional profiling identifies a role for BrlA in the response to nitrogen depletion and for StuA in the regulation of secondary metabolite clusters in *Aspergillus fumigatus*. Eukaryot Cell 8:104–115. doi:10.1128/EC.00265-08. 19028996 PMC2620752

[31] Baker CL, Loros JJ, Dunlap JC 2012 The circadian clock of *Neurospora crassa*. FEMS Microbiol Rev 36:95–110. doi:10.1111/j.1574-6976.2011.00288.x. 21707668 PMC3203324

[32] Chen CH, Dunlap JC, Loros JJ 2010 *Neurospora* illuminates fungal photoreception. Fungal Genet Biol 47:922–929. doi:10.1016/j.fgb.2010.07.005. 20637887 PMC3649881

[33] Dunlap JC, Loros JJ 2006 How fungi keep time: circadian system in *Neurospora* and other fungi. Curr Opin Microbiol 9:579–587. doi:10.1016/j.mib.2006.10.008. 17064954

[34] Greene AV, Keller N, Haas H, Bell-Pedersen D 2003 A circadian oscillator in *Aspergillus* spp. regulates daily development and gene expression. Eukaryot Cell 2:231–237. doi:10.1128/EC.2.2.231-237.2003. 12684372 PMC154850

[35] Bhabhra R, Miley MD, Mylonakis E, Boettner D, Fortwendel J, Panepinto JC, Postow M, Rhodes JC, Askew DS 2004 Disruption of the *Aspergillus fumigatus* gene encoding nucleolar protein CgrA impairs thermotolerant growth and reduces virulence. Infect Immun 72:4731–4740. doi:10.1128/IAI.72.8.4731-4740.2004. 15271935 PMC470587

[36] Willger SD, Puttikamonkul S, Kim KH, Burritt JB, Grahl N, Metzler LJ, Barbuch R, Bard M, Lawrence CB, Cramer RA Jr. 2008 A sterol-regulatory element binding protein is required for cell polarity, hypoxia adaptation, azole drug resistance, and virulence in *Aspergillus fumigatus*. PLoS Pathog 4:e1000200. doi:10.1371/journal.ppat.1000200. 18989462 PMC2572145

[37] Hagiwara D, Takahashi H, Watanabe A, Takahashi-Nakaguchi A, Kawamoto S, Kamei K, Gonoi T 2014 Whole-genome comparison of *Aspergillus fumigatus* strains serially isolated from patients with aspergillosis. J Clin Microbiol 52:4202–4209. doi:10.1128/JCM.01105-14. 25232160 PMC4313286

[38] Abdolrasouli A, Rhodes J, Beale MA, Hagen F, Rogers TR, Chowdhary A, Meis JF, Armstrong-James D, Fisher MC 2015 Genomic context of azole resistance mutations in *Aspergillus fumigatus* determined using whole-genome sequencing. mBio 6:e00536. doi:10.1128/mBio.00536-15. 26037120 PMC4453006

[39] Lazzarini C, Esposto MC, Prigitano A, Cogliati M, De Lorenzis G, Tortorano AM 2016 Azole resistance in *Aspergillus fumigatus* clinical isolates from an Italian culture collection. Antimicrob Agents Chemother 60:682–685. doi:10.1128/AAC.02234-15. 26552980 PMC4704201

[40] Luque EM, Gutiérrez G, Navarro-Sampedro L, Olmedo M, Rodríguez-Romero J, Ruger-Herreros C, Tagua VG, Corrochano LM 2012 A relationship between carotenoid accumulation and the distribution of species of the fungus *Neurospora* in Spain. PLoS One 7:e33658. doi:10.1371/journal.pone.0033658. 22448263 PMC3309001

[41] Michael TP, Park S, Kim TS, Booth J, Byer A, Sun Q, Chory J, Lee K 2007 Simple sequence repeats provide a substrate for phenotypic variation in the *Neurospora crassa* circadian clock. PLoS One 2:e795. doi:10.1371/journal.pone.0000795. 17726525 PMC1949147

[42] Tan KK, Epton HAS 1973 Effect of light on the growth and sporulation of *Botrytis cinerea*. Trans Br Mycol Soc 61:145–157. doi:10.1016/S0007-1536(73)80096-8.

[43] Suzuki Y, Oda Y 1979 Inhibitory loci of both blue and near ultraviolet lights on lateral-type sclerotial development in *Botrytis cinerea*. Jpn J Phytopathol 45:54–61. doi:10.3186/jjphytopath.45.54.

[44] Schumacher J, Pradier JM, Simon A, Traeger S, Moraga J, Collado IG, Viaud M, Tudzynski B 2012 Natural variation in the VELVET gene *bcvel1* affects virulence and light-dependent differentiation in *Botrytis cinerea*. PLoS One 7:e47840. doi:10.1371/journal.pone.0047840. 23118899 PMC3485325

[45] Cramer RA Jr, Perfect BZ, Pinchai N, Park S, Perlin DS, Asfaw YG, Heitman J, Perfect JR, Steinbach WJ 2008 Calcineurin target CrzA regulates conidial germination, hyphal growth, and pathogenesis of *Aspergillus fumigatus*. Eukaryot Cell 7:1085–1097. doi:10.1128/EC.00086-08. 18456861 PMC2446674

[46] Soriani FM, Malavazi I, da Silva Ferreira ME, Savoldi M, Von Zeska Kress MR, de Souza Goldman MH, Loss O, Bignell E, Goldman GH 2008 Functional characterization of the Aspergillus fumigatus CRZ1 homologue, CrzA. Mol Microbiol 67:1274–1291. doi:10.1111/j.1365-2958.2008.06122. 18298443

[47] Campbell CC, Berliner MD 1973 Virulence differences in mice of type A and B *Histoplasma capsulatum* yeasts grown in continuous light and total darkness. Infect Immun 8:677–678. 4742977 10.1128/iai.8.4.677-678.1973 PMC422910

[48] Cove DJ 1966 The induction and repression of nitrate reductase in the fungus *Aspergillus nidulans*. Biochim Biophys Acta 113:51–56. doi:10.1016/S0926-6593(66)80120-0. 5940632

