# Supplementary figures and images for: Aspergillus fumigatus Photobiology Illuminates the Marked Heterogeneity between Isolates
Source: mBio. 2016 Sep 20;7(5):e01517-16. doi: 10.1128/mBio.01517-16 (PMC5030361; doi:10.1128/mBio.01517-16)

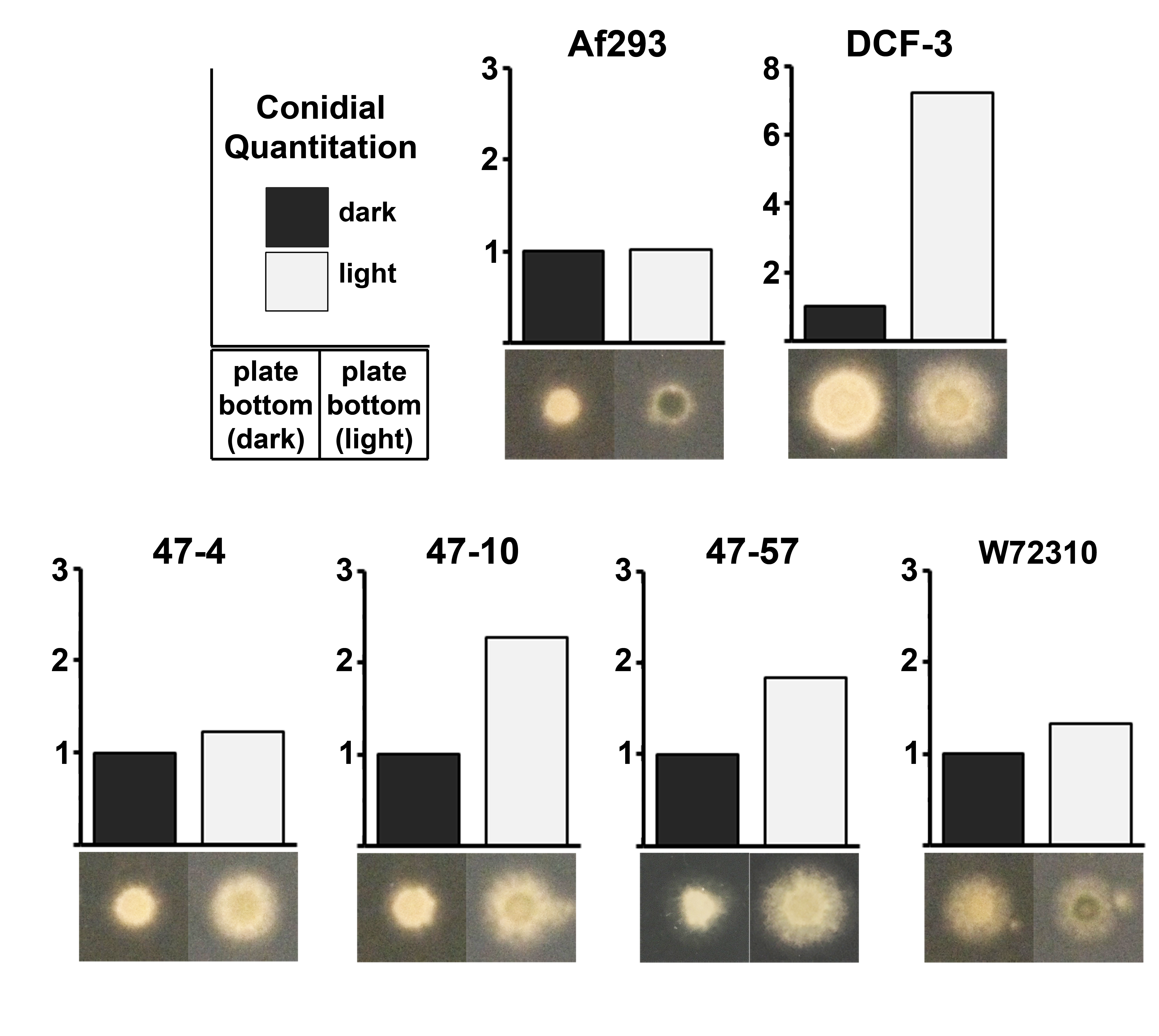

Supplement: Figure S1 — Photopigmentation and photoconidiation responses of additional A. fumigatus isolates. Conidia of the indicated strain were point inoculated onto GMM (plate pictures) or RPMI 1640 (graphs) and incubated for 48 h either in constant darkness or under constant white light illumination. Conidiation values are normalized to the dark-grown sample of the indicated strain. Enumeration of conidia was performed in triplicate, and data were statistically analyzed by Student’s t test (*, P ≤ 0.05). Download [file mbo004162992sf1.tif]

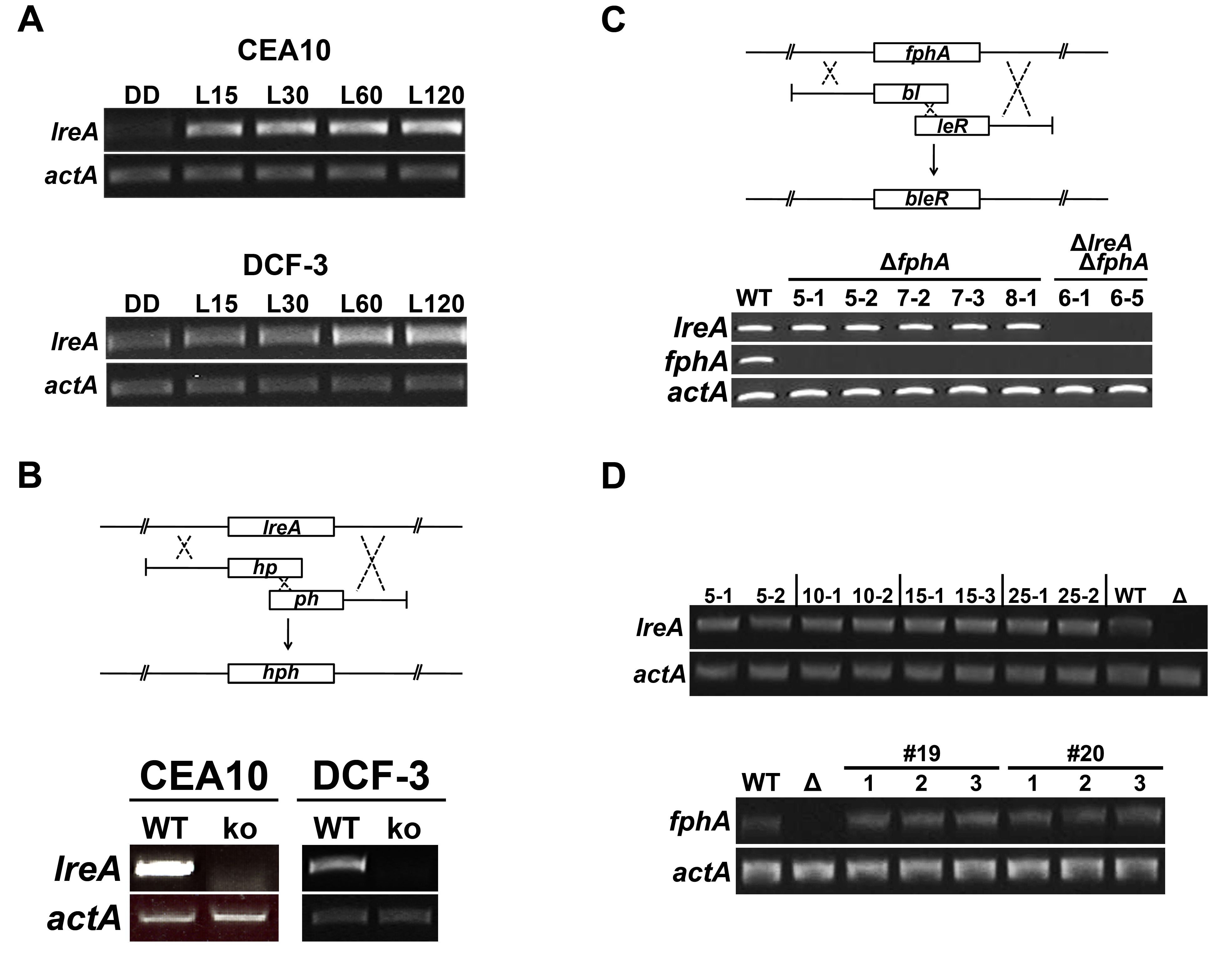

Supplement: Figure S3 — (A) RT-PCR analysis of lreA expression. Strains were incubated in the dark for 48 h and then transferred to constant white light illumination for the indicated time points. (B) Schematic depiction of the split-marker deletion strategy of lreA is shown as well as an RT-PCR demonstrating loss of the lreA transcript in the putative knockouts. hph, hygromycin phosphotransferase gene. (C) Schematic depiction of the split-marker deletion strategy of fphA is shown as well as an RT-PCR demonstrating loss of the fphA transcript in either the CEA10 WT or ΔlreA background. (D) RT-PCR demonstrating the transcript of either lreA (top) or fphA (bottom) in the respective complemented strain. Download [file mbo004162992sf3.tif]

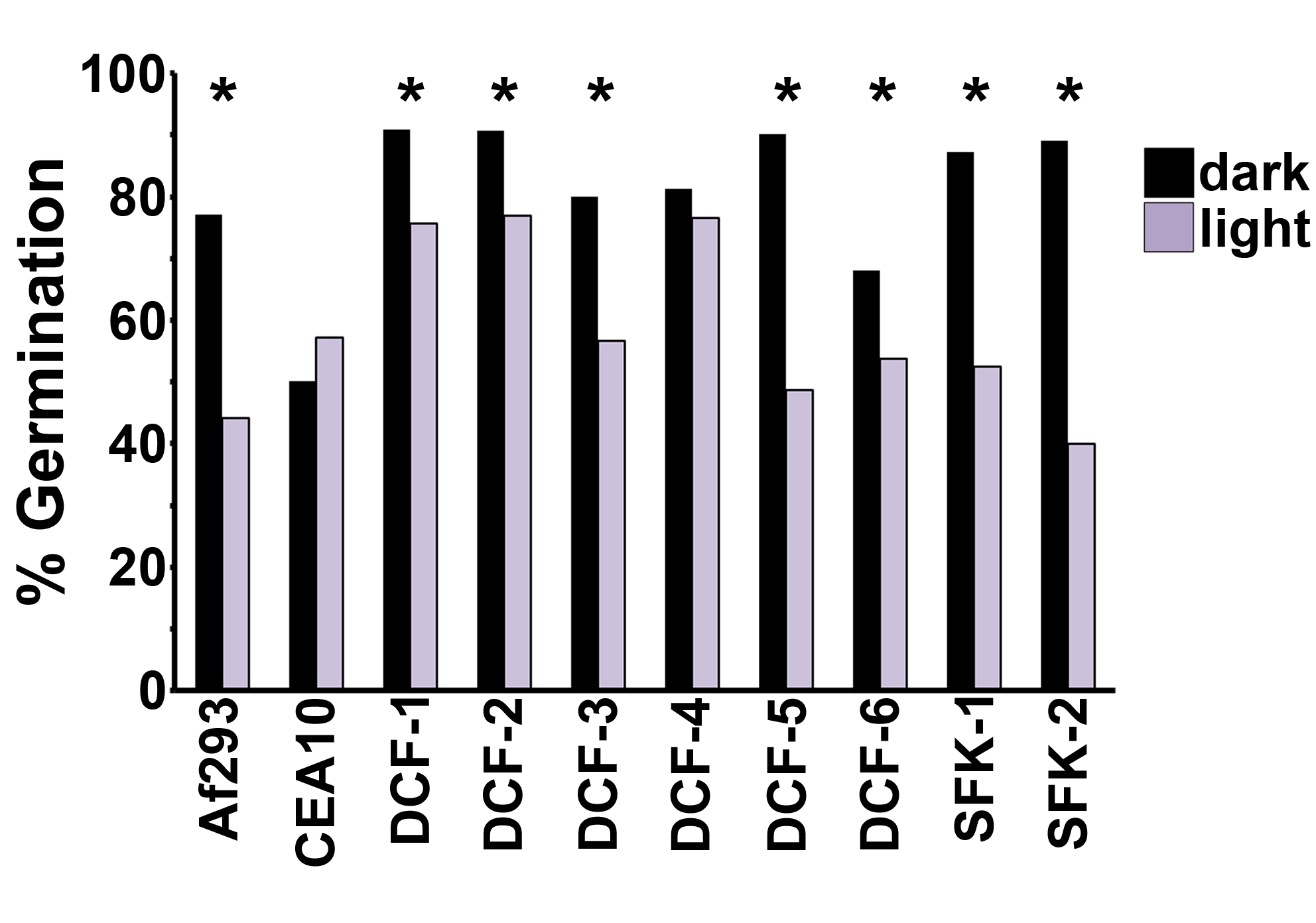

Supplement: Figure S4 — Light decreases germination rates in most A. fumigatus isolates. Conidia were inoculated into GMM and incubated for 8 h either in constant darkness or under constant white light illumination. A minimum of 300 conidia were scored for the presence or absence of a germ tube in each group, and light and dark samples were compared by the chi-square test (*, P ≤ 0.05). Download [file mbo004162992sf4.tif]

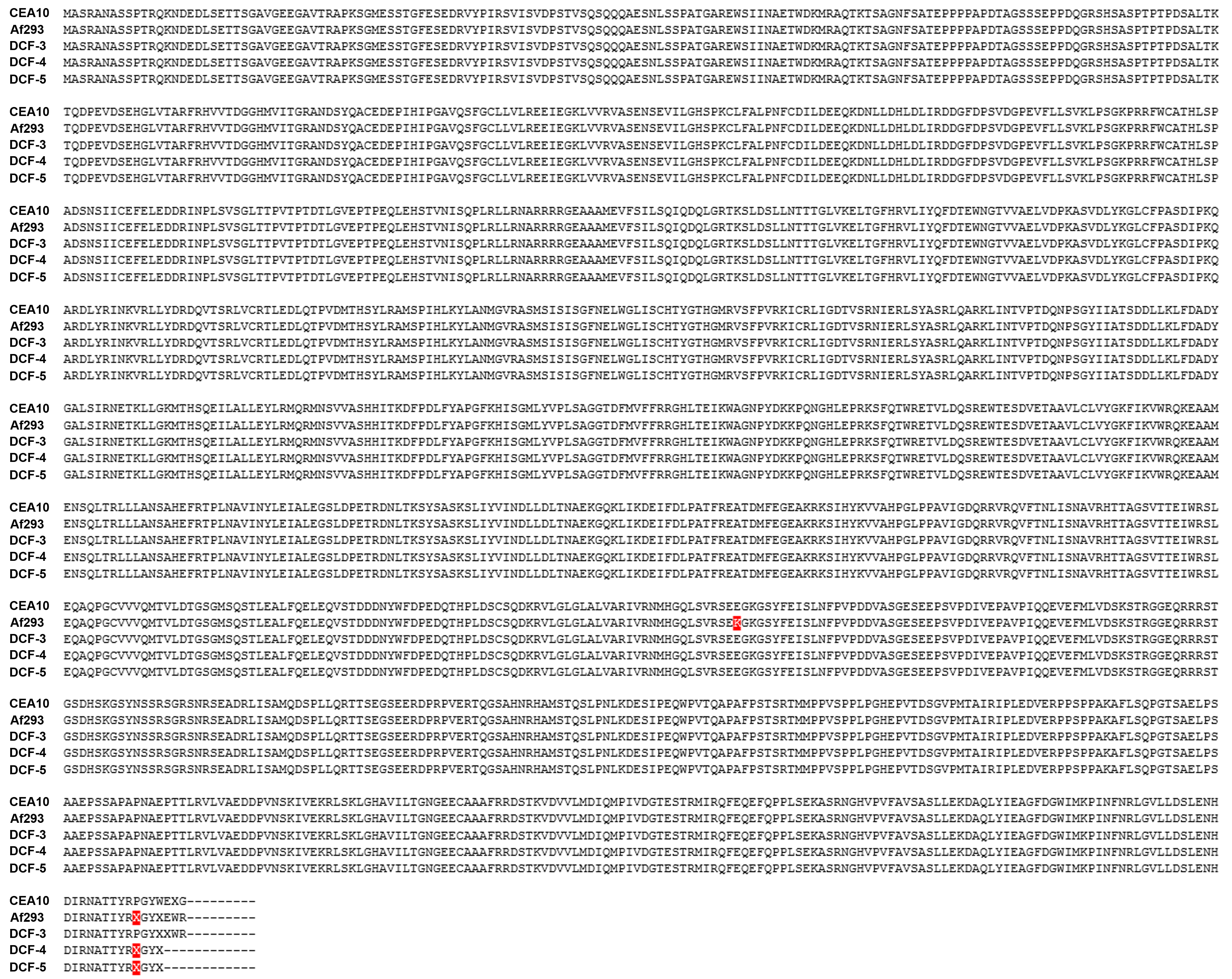

Supplement: Figure S5 — FphA protein sequence alignment based on Sanger sequencing from the indicated isolates. Download [file mbo004162992sf5.tif]

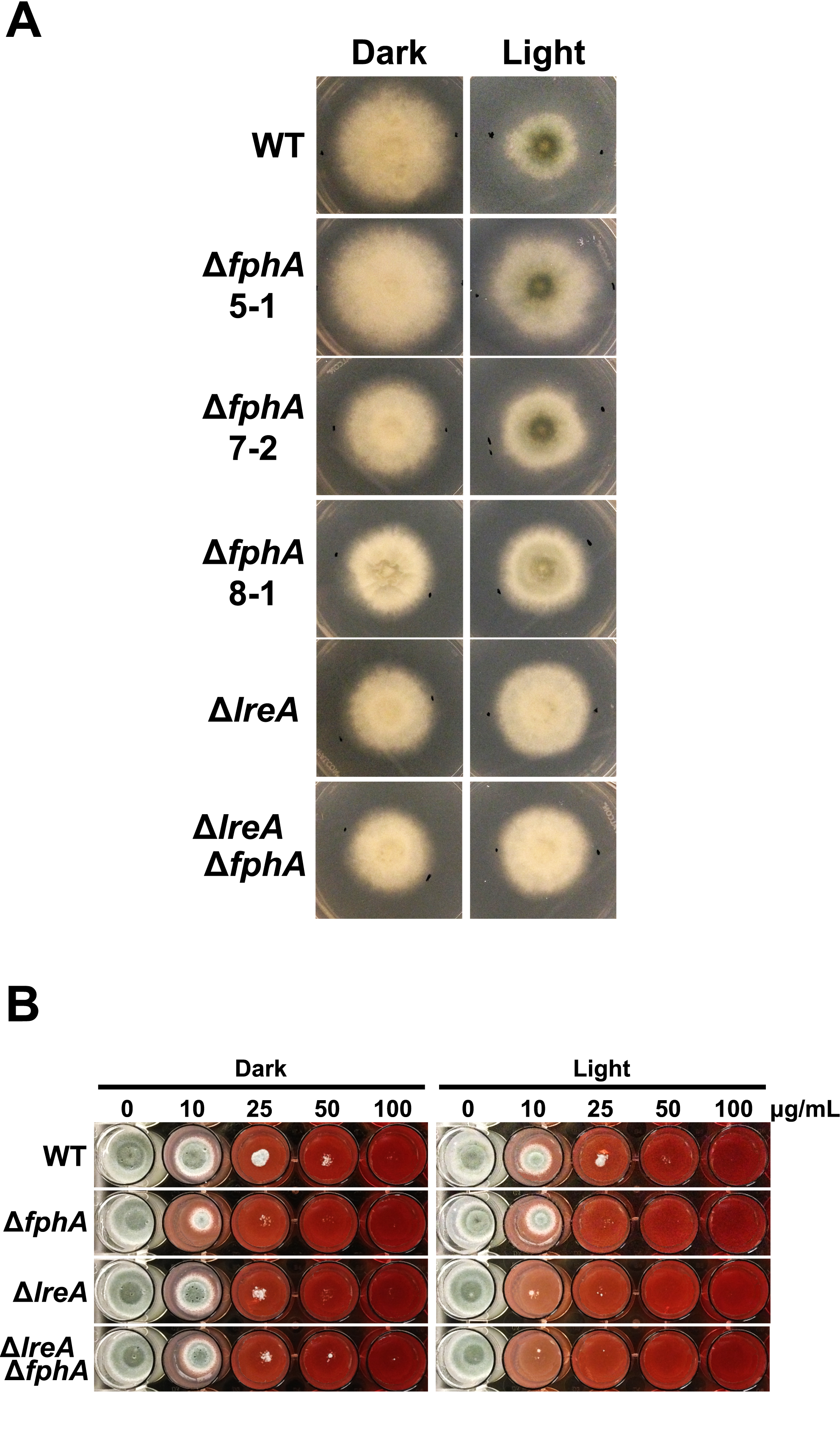

Supplement: Figure S6 — Additional phenotypes of the CEA10 photoreceptor mutants. (A) Pigmentation. Conidia were point inoculated into GMM plates and incubated at 37°C for 48 h either in constant darkness or under constant illumination (blue plus red LEDs). Several independent isolates of the ΔfphA mutation are shown and are distinguished numerically (e.g., 5-1, 7-2). (B) Congo red sensitivity. Conidia were point inoculated onto GMM plates supplemented with the indicated concentration of Congo red. Plates were then incubated for 48 h at 37°C for 48 h either in constant darkness or under constant illumination (blue plus red LEDs). Download [file mbo004162992sf6.tif]

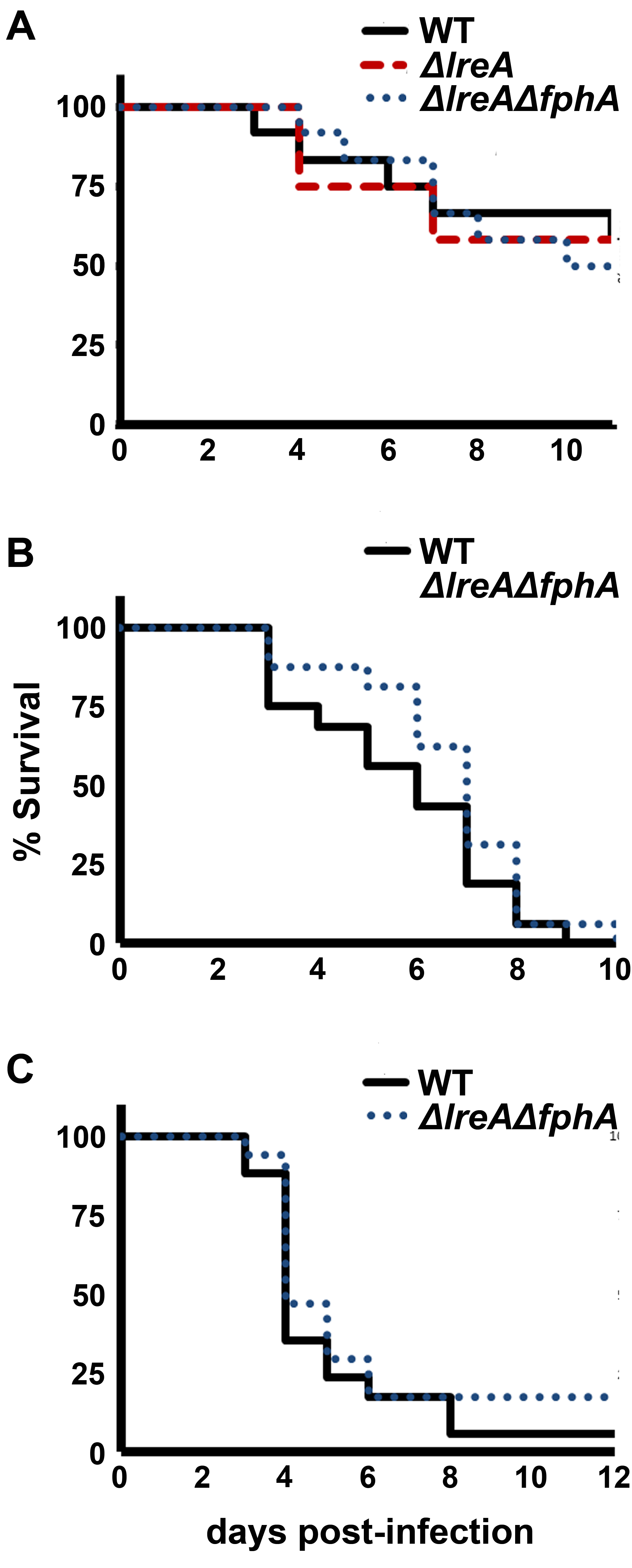

Supplement: Figure S7 — Deletion of lreA in the Af293 background does not attenuate virulence. (A) Single-dose steroid model. Groups of 12 CD-1 mice were immunosuppressed with Kenalog-10 (day −1) and inoculated intranasally with 2.0 × 10^6^ conidia of the indicated genotype. (B) Multidose steroid model. Groups of 16 CD-1 mice were immunosuppressed with two doses of Kenalog-10 (days −1 and +3) and inoculated intranasally with 1.5 × 10^6^ conidia of the indicated genotypes. Survival curves are not statistically different (P = 0.22, log rank test). (C) Chemotherapy (neutropenia model). Groups of 17 CD-1 mice were immunosuppressed with cyclophosphamide (days −2 and +3) and Kenalog-10 (day −1) and inoculated intranasally with 2.0 × 10^6^ conidia of the indicated genotypes. Survival curves are not statistically different (P = 0.4, log rank test). Download [file mbo004162992sf7.tif]
