# Supplementary material for: Aspergillus fumigatus Photobiology Illuminates the Marked Heterogeneity between Isolates
Source: mBio. 2016 Sep 20;7(5):e01517-16. doi: 10.1128/mBio.01517-16 (PMC5030361; doi:10.1128/mBio.01517-16)
Supplement: Table S1 — Strains used in this study [file mbo004162992st1.docx]

| **Strain name** | **Source** | **Description** | **ref** |
| --- | --- | --- | --- |
| Af293 (A1100) | FGSC | invasive aspergillosis isolate |  |
| CEA10 (A1163) | FGSC | invasive aspergillosis isolate |  |
| H237 | UC (USA) | invasive aspergillosis isolate |  |
| DCF-1 (42-2465) | DHMC (USA) | CF sputum isolate | this study |
| DCF-2 (43-2135) | DHMC (USA) | CF sputum isolate | this study |
| DCF-3 (43-3066) | DHMC (USA) | CF sputum isolate | this study |
| DCF-4 (63-3447) | DHMC (USA) | CF sputum isolate | this study |
| DCF-5 (63-3472) | DHMC (USA) | CF sputum isolate | this study |
| DCF-6 (71-3123) | DHMC (USA) | CF sputum isolate | this study |
| SFK-1 (Af09-0584) | UCSF (USA) | keratitis isolate, UCSF | this study |
| SFK-2 (Af09-0868) | UCSF (USA) | keratitis isolate, UCSF | this study |
| 47-4 (Af250) | Salford Royal (UK) | environmental isolate (hospital) | this study |
| 47-10 (Af221) | (New Zealand) | environmental isolate (wood) | this study |
| 47-57 (AFIR957) | (Ireland) | environmental isolate (air) | this study |
| W72310 | MSK (USA) | CF sputum isolate | this study |

FGSC; Fungal Genetics Stock Center, Manhattan, KS, USA

UCMC; University of Cincinnati Medical Center, Cincinnati, OH, USA

DHMC; Dartmouth Hitchcock Medical Center, Lebanon, NH, USA

UCSF; University of San Francisco Medical Center, San Francisco, CA, USA

MSK; Memorial Sloan Kettering Medical Center, New York City, NY, USA
