# Supplementary material for: Aspergillus fumigatus Photobiology Illuminates the Marked Heterogeneity between Isolates
Source: mBio. 2016 Sep 20;7(5):e01517-16. doi: 10.1128/mBio.01517-16 (PMC5030361; doi:10.1128/mBio.01517-16)
Supplement: Table S2 — Summary of photoresponsive behaviors in the analyzed isolates. Photopigmenting isolates are shaded. [file mbo004162992st2.docx]

| Strain | Light effect on: | Germination | Pigmentation | Conidiation |
| --- | --- | --- | --- | --- |
| Af293 |  | repression | **strong induction** | repression |
| CEA10 |  | no effect | **strong induction** | no effect |
| H237 |  | repression | no effect | **induction** |
| DCF-1 |  | repression | **strong induction** | no effect |
| DCF-2 |  | repression | no effect | **induction** |
| DCF-3 |  | repression | no effect | **induction** |
| DCF-4 |  | no effect | no effect | **induction** |
| DCF-5 |  | repression | no effect | **induction** |
| DCF-6 |  | repression | no effect | **induction** |
| SFK-1 |  | repression | weak induction | **induction** |
| SFK-2 |  | repression | weak induction | **induction** |
| 47-4 |  | not tested | no effect | no effect |
| 47-10 |  | not tested | no effect | **induction** |
| 47-57 |  | not tested | no effect | **weak** |
| W72310 |  | not tested | weak induction | no effect |
